# On the Use of Polymer-Based Composites for the Creation of Optical Sensors: A Review

**DOI:** 10.3390/polym14204448

**Published:** 2022-10-21

**Authors:** Pavel Melnikov, Alexander Bobrov, Yuriy Marfin

**Affiliations:** 1 M. V. Lomonosov Institute of Fine Chemical Technologies, MIREA—Russian Technological University, 119571 Moscow, Russia; 2 Department of Inorganic Chemistry, Ivanovo State University of Chemistry and Technology, Sheremetevsky pr., 10, 153010 Ivanovo, Russia; 3 Pacific National University, 136 Tikhookeanskaya Street, 680035 Khabarovsk, Russia

**Keywords:** polymer-based nanocomposites, polymer matrix enhancement, surface modification, optical sensor, biocomposite materials, aza-BODIPY, molecular design, fluorescence, absorption

## Abstract

Polymers are widely used in many areas, but often their individual properties are not sufficient for use in certain applications. One of the solutions is the creation of polymer-based composites and nanocomposites. In such materials, in order to improve their properties, nanoscale particles (at least in one dimension) are dispersed in the polymer matrix. These properties include increased mechanical strength and durability, the ability to create a developed inner surface, adjustable thermal and electrical conductivity, and many others. The materials created can have a wide range of applications, such as biomimetic materials and technologies, smart materials, renewable energy sources, packaging, etc. This article reviews the usage of composites as a matrix for the optical sensors and biosensors. It highlights several methods that have been used to enhance performance and properties by optimizing the filler. It shows the main methods of combining indicator dyes with the material of the sensor matrix. Furthermore, the role of co-fillers or a hybrid filler in a polymer composite system is discussed, revealing the great potential and prospect of such matrixes in the field of fine properties tuning for advanced applications.

## 1. Introduction

Measurement and control of the composition of aquatic environments, soil and air, as well as the quality assurance of various products produced by human is impossible without the use of analytical systems, and their improvement is a necessary permanent task. The latest trend is the development of smart materials that respond to given influences from external sources through physical or chemical reaction, which leads to a change in the characteristics of the material [1]. The smart polymer-based materials play an increasingly important role [2]. Among the possible sources of an analytical signal, one can distinguish pH, enzyme, redox, temperature, electric and magnetic fields, etc. At the same time, electrochemical and optical measuring systems occupy an overwhelming position among practical applications of the transducer type [3]. It is important to note that, for example, when measuring oxygen, there is a competition between the mentioned detection methods, and optical systems are gradually replacing electrochemical ones [4]. The main reason is that the optical method is not limited to measurements at a single point but allows imaging and intracellular measurements by means of nanoparticles with indicator dye, and fiber optic microsensors greatly facilitate their integration into research or industrial equipment. Among the practical applications are plant and animal physiology research, marine sciences, clinical chemistry, biotechnology and chemical industry [4].

Polymer-based sensors are of increasing interest because the ingredients used could be easily processed and could be matched for biocompatibility [5,6,7]. The steady increase in publications on the topic and investment in this area of research are proof that such technologies are among the most promising at the moment [1,8]. Conducting polymers, hydrogels, molecularly imprinted polymers (MIP), and composites and nanocomposites are the common polymer-based materials used in sensing devices [9,10,11,12]. The latter are the most interesting, as they have the widest scope for customizing the properties of the system being created [13,14,15]. On the one hand, the creation of such polymer-based composite systems can improve the molecular recognition, where the polymer serves as the matrix for the immobilization of various functional groups (dyes, luminescent nanofillers, etc.) and makes it possible to detect target analytes [16,17]. Another important advantage of polymer-based sensors is the ability to tune their physical/chemical properties so that their resistance to degradation, biodegradability and flexibility can be modified [1,18]. The increasing opportunities for recycling and upcycling of polymers should also be mentioned [19,20].

The aim of this review is to highlight the leading trends, challenges and approaches to the creation of polymer-based composite materials for optical sensors with controlled properties. The main methods of combining indicator dyes with the material of the sensor matrix are also shown. Furthermore, the role of co-fillers or a hybrid filler in a polymer composite system is also discussed, revealing the great potential and prospect of such matrixes in the field of fine properties tuning for particular applications. Examples of modern advances in polymer-based optical sensors for the detection of various analytes in environmental objects and biological systems are also given.

## 2. Methods for Immobilization of Luminophores in a Polymeric Matrix

Luminophores (a chemical compound that has luminescent properties, i.e., fluorescence or phosphorescence) can be used in various fields of science. But their practical application is hampered by their instability to UV radiation, high temperature, chemicals like acids, etc. In order to maintain the practical properties of the luminophores and keep them from detrimental effects, they are placed in various polymer matrices, where their application becomes easier compared to solutions [21,22,23,24,25].

Dye-doped thin films, which can be used as solid-state laser limiters and tunable laser systems, are the most widely used today, surpassing dye solutions in their stability and optical characteristics [26,27].

A new application of hybrid materials containing optically active materials is the obtainment of luminescent solar concentrators [28,29]—coatings on the surface of solar cells that shift solar radiation spectra into the area of most effective absorption by the solar cell. The authors of [30] report about obtaining hybrid materials containing a mixture of chromophores based on BODIPY, absorbing in different regions of the spectrum, which allowed to increase the efficiency of solar cells due to a higher degree of conversion.

The use of hybrid materials as components of optical, primarily, laser systems impose a number of requirements regarding the composition and morphology of hybrid materials: preservation of practically useful optical properties of dye in the hybrid material, effective dissipation of generated heat (thin-film materials are quite effective), etc.

Another application of hybrid materials is the creation of highly effective fluorescent tags and sensors; in this case, the material is used in the form of a powder. Created tags have a number of advantages: one microgranule of hybrid material can contain a significant number of dye molecules, which increases the detection limit of the sensor when such a microgranule is attached to the binding site. The matrix of the hybrid material protects the dye molecules from the interaction with fluorescence quenching molecules. The creation of hybrid materials containing several chromophore compounds at once (multichromophore materials) makes it possible to expand the use of fluorescence spectroscopy for biomedical purposes [31].

When designing materials for medical and molecular biosystems sensing applications, such as in vivo pH determination, it is advisable to consider nano-objects [32,33]. Some functional nanomaterials based on boron dipyrrin luminophores have been developed and can be used agents in PDT and imaging in biological systems. Thus, polymeric nanoparticles are of considerable interest for medical diagnostic and therapeutic applications [34,35]. For example, drugs delivering system (nanoscale micelles or vesicles) can be synthesized by self-assembly of amphiphilic copolymers in aqueous solution. Recent research has proven that such delivering systems with antitumor drugs provide more effective tumor destruction compared to the free drug [36]. Fluorescent dyes also can be used as models to prove success of encapsulation and allow monitor the drug delivery in vivo. Some BODIPY dyes have already established themselves as fluorescent probes, markers in living systems, and components of targeted drug delivery [37,38,39,40].

**Figure 1:**
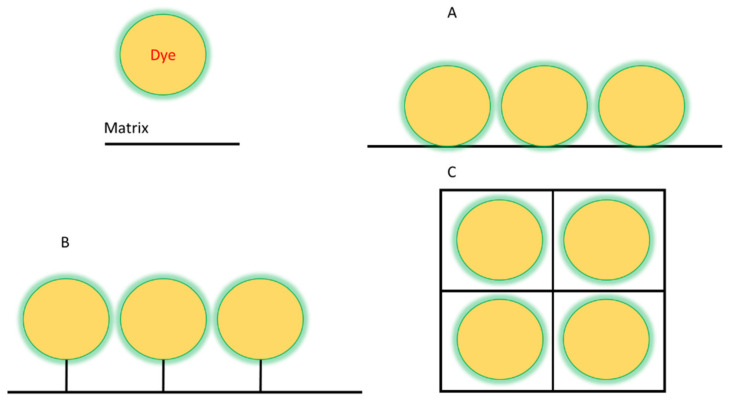
Scheme of the various dye immobilization methods: (**A**)—adsorption, (**B**)—covalent binding, (**C**)—encapsulation.

Dye immobilization can be achieved by two different methods: physical and chemical. The main difference between these methods lies in the strength of the bond between the matrix and the dye. If they are weak interactions, in the form of hydrogen bonds, etc., then it is a physical method. In the case of covalent bonding, it is the chemical method [41,42,43,44]. Schematically shown in Figure 1. The methods of binding matrix to dye almost the same as in supramolecules between their two or more parts [45].

A brief discussion of above-mentioned methods is summarized below.

### 2.1. Non-Covalent Binding of Dye Molecule to Preformed Polymer (Adsorption)

**Figure 2:**
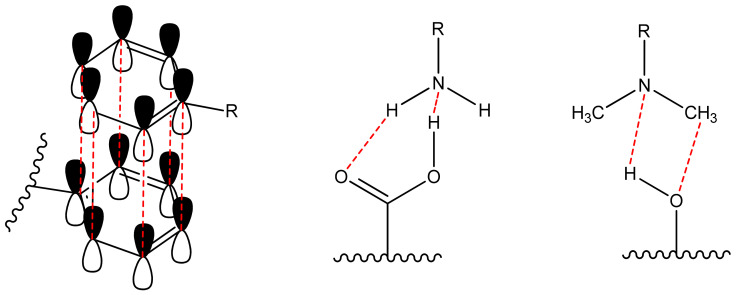
Scheme of non-covalent bonding between dye and matrix (π-π stacking and hydrogen bonds).

Non-covalent (physical) binding can take place through various types of interactions, such as ion-dipole, dipole-dipole interactions, complex formation, hydrogen bonds, π-π-stacking, etc. (Figure 2). There are many polar substituents that contribute to the formation of dipole interactions with the corresponding substrates. Such matrices include: among the organic ones most popular are oligo-polysaccharides [46,47], cellulose [48,49,50,51], polymethylmethacrylate (PMMA) [52,53,54,55], polyvinyl chloride (PVC) [11,56]; among inorganic ones most popular are silica and other polymeric oxides obtained by sol-gel synthesis [57,58,59,60,61,62]. On the other hand, the luminophore must have the following groups (one or several): H-, hydroxo-, amino-, nitro-, sulpho-, mercapto- and groups comprising from atoms with an unsplit electron pair and capable of forming a donor-acceptor bond; phenyl and other cyclically conjugated molecules to form π-π stacking.

The absence of these groups also allows immobilization of the dye in the matrix pores through intermolecular interactions and Van-der-Waals forces, i.e., simple sorption.

The most common and popular usage of materials nowadays is the sensorics. Materials could be used as sensors for determining pH, the polarity of the environment and for detecting specific substances and molecules ions. For example, using matrices of silica and its organo-modified counterpart, a pH sensor can be produced using the sol-gel method. And depending on the choice of matrix, the nature of the sensor response may vary or not appear at all [61]. PMMA matrices with BODIPY can be used to produce sensors for polarity reversal of the environment [62]. Ethyl cellulose matrices with BODIPY or bis-dipyrrin complexes can be used for the some gases detection in micro quantities in air [48,49,63]. It should also be noted that sorption increases the threshold for aggregation, i.e., more dye can be placed in the matrix without forming aggregates [64].

### 2.2. Covalent Binding of Dye Molecule to Polymer

One of chemical method involves the formation of covalent bonds between the dye and the matrix. One or several of dye’s substituents which are not responsible for any optical or other important activity are linked to matrix. Such substitutes include groups like carboxy, phenolic hydroxyl, amino, indolyl, hydroxy, etc. [65,66,67,68,69,70,71,72]. One of requirements of such binding reaction is that after it there should not be a loss of fluorescent, sensory, or other activity, and the core of the dye must be unaffected.

Binding between the luminophore and the matrix can be achieved either via covalent binding to the luminophore core or via binding to a long aliphatic substituent called a spacer. By using a spacer, you can achieve greater mobility of the dye molecule. So, there is usually a reactive group at the end of the spacer that reacts with another group on the dye to form a covalent bond. These are the same groups as mentioned earlier.

**Figure 3:**
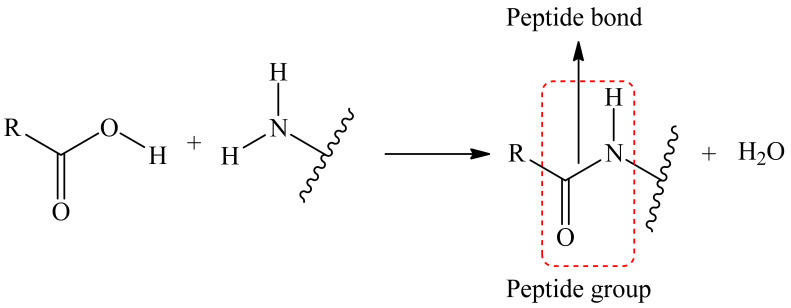
Scheme of the peptide bonding process.

The reaction to form a covalent bond can be anything; the main thing to keep in mind is that the conditions for this reaction must not destroy the matrix as well as the dye itself. As an example, we can take the reaction where the reaction of the carboxyl tail (matrix/dye) with an amino group (dye/matrix) forms a covalent bond between two substances, i.e., a peptide bond is formed (Figure 3).

One method of producing a covalently bonded hybrid material is to bind a dye to a precursor of the future polymer. For example, the authors [73] proposed to add a precursor for sol-gel synthesis to the beta-substitute of aza-BODIPY, where the inorganic polymer was formed by hydrolysis and polycondensation reactions. The resulting inorganic hybrid material was eventually used as power limiting filters at telecommunication wavelengths.

Materials obtained by covalent binding are used to increase the effectiveness of photodynamic therapy (by covalent bonding of BODIPY through hydroxyl group to DND/Pc hybrid) [74], in block materials with AIE effect [75], solar cells [76,77], FET devices [78], material for sensing and removal Hg^2+^ and Fe^3+^ (was synthesized by click reaction between NH_2_/C≡C (chitosan(CS)/dye)) [79], sensors for guanine and adenine in PBS (SWCNT matrix) [80], pH and thermo responsive nano probe (rGO matrix) [81], CO_2_ adsorption (by preparing porous luminescent polymer) [82], self-healing gels [83], wastewater treatment [84].

**Figure 4:**
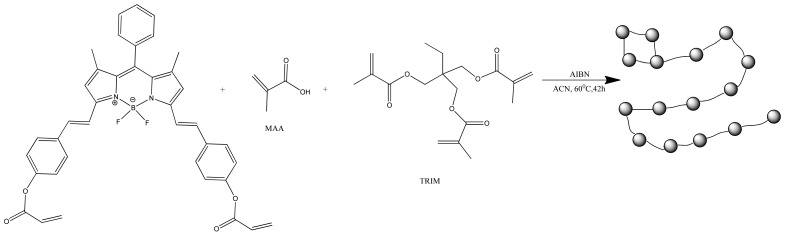
Synthesis of the fluorescent polymer nanoparticles (Reprinted from Hoji, A., et al., Syntheses of BODIPY-incorporated polymer nanoparticles with strong fluorescence and water compatibility. Eur. Polym. J. 2020, with permission from Elsevier).

**Figure 5:**
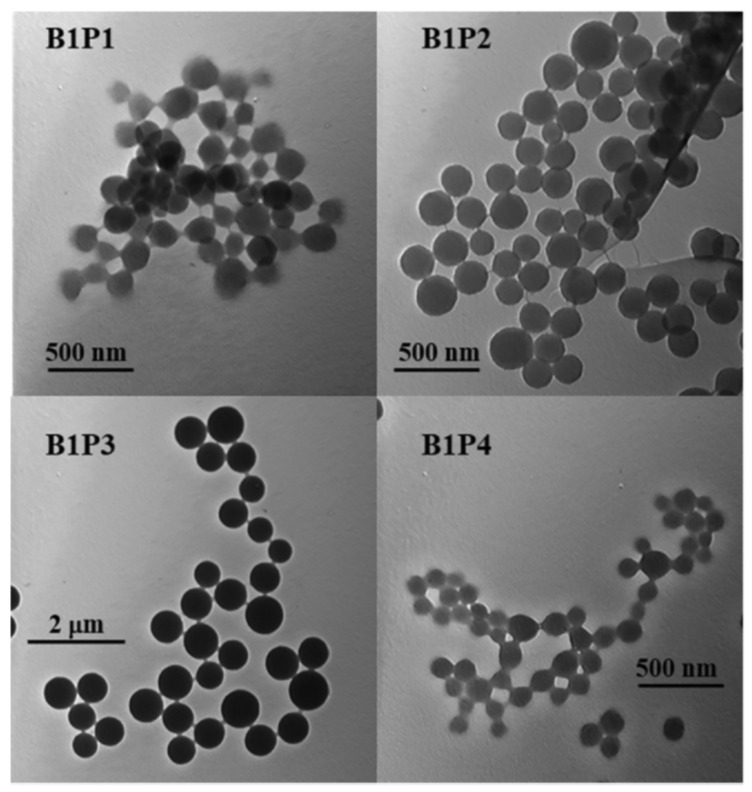
Transmission electronic microscopy (TEM) images of polymers B1P1, B1P2, B1P3 and B1P4 synthesized by precipitation polymerization. (Reprinted from Hoji, A., et al., Syntheses of BODIPY-incorporated polymer nanoparticles with strong fluorescence and water compatibility. Eur. Polym. J. 2020, with permission from Elsevier).

With covalent binding, some of the properties of the luminophore can be changed by the properties of the matrix itself. Scientists have produced a water-soluble polymeric hybrid material consisting of a BODIPY luminophore (insoluble in water) and a monomer containing methacrylic acid (MAA, water soluble). The hybrid material was used as a sensor for Fe^3+^ in water. This can also include an example with the production of polymeric water-soluble nanoparticles from a dye and a monomer [85]. A scheme and TEM of the samples are shown in Figure 4 and Figure 5, respectively.

This method also includes the group of coordination polymers, where the luminophore is a part of the polymer chain itself. In this case, first a monomer containing a dye is obtained, from which, after a polymerisation reaction, the final fluorescent polymer is obtained. Thus, in [86] a coordination polymer with BODIPY in its chain was obtained. The obtained polymer was used for detoxification by photochemical generation of singlet oxygen. A BODIPY-diketopyrrolopyrrole conjugate polymer was obtained by the authors [87], obtained material was used for photothermal tumor ablation.

### 2.3. Encapsulation

One method of immobilization that can be either physical or chemical, depending on your needs, is encapsulation [88]. Dye encapsulation in gels or fibers is a convenient method for use in processes involving low molecular weight substances and products. 

Dye-encapsulated nanoparticles were prepared by the authors [89]. The particles were synthesized from poly(D,L-lactide-co-glycolide)(PLGA) and dye in acetonitrile, and the resulting mixture was added to phosphate buffer from a micropipette after shaking to obtain the final product. The material obtained in this way was used to investigate the process of the uncontrolled release of encapsulated drugs in living cells. Another example is the encapsulation of luminophores in polymeric nanoparticles and applications in the field of cancer cell imaging [90].

Dyes could be entrapped in silica-based core/shell nanoparticles [91], polyurethane hydrogels [92], MOF’s(metal-organic frameworks) [93], liposomes [94], niosomes [95], bacterial biofilms [96], nanoparticles [97], aerogels [98], and other matrixes.

### 2.4. Choice of Matrix for Immobilization

Matrix characteristics are important in determining the efficiency of systems with immobilized dyes. Ideal matrix should have resistance to mechanical destruction, inertness toward dye, biocompatibility (if needed), and resistance to UV light, resistance towards acids or alkali and small purchase/synthesis price. Some of the polymers like cellulose, silica, aerogels, poly(methyl methacrylate) and other organic and inorganic polymer materials are commonly used as matrixes. Because they possess good mechanical stability, moreover they can be modified easily. Silica-based matrices are the most suitable matrices for dye immobilization in industrial manufacturing of products for lasers and solar panels and for research purposes because of their high resistance to UV, acids and mechanical destruction.

## 3. Tuning the Properties of Composites through the Introduction of Various Fillers

**Figure 6:**
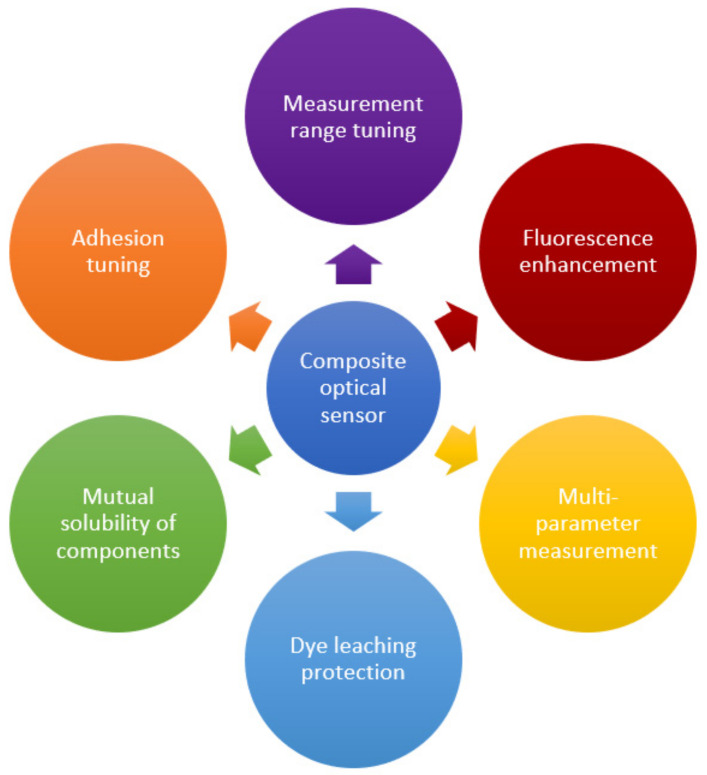
The main qualitative improvements in optical sensors achieved through the creation of composite materials.

Polymer-based composites and nanocomposites are hybrid materials obtained by the dispersion of nanosized particles (at least in one dimension) in an organic polymer matrix to improve its properties. Among the effects achieved, one can mention the possibility of creating a developed inner surface, mechanical strength, adjustable electrical and thermal conductivity, and many others. A wide variety of applications of created materials are known, including biomimetic technologies, renewable energy sources, smart materials, packaging, etc. [5,6,13,99]. This section discusses the main options for tuning of polymer-based nanocomposites used in optical sensors. Table 1 provides a summary of some examples of polymer nanocomposites’ use, as well as the benefits of the nanofiller incorporation. The main qualitative improvements achieved through the transition to composite materials are summarized in Figure 6. The following section presents the main ways to achieve these advances. The material is grouped according to the type of the filler used to create the composite, since it usually has a decisive influence on the properties and can simultaneously achieve several goals indicated in Figure 6. Options for improving the host polymer matrix itself and improving the mutual adhesion of the components to stabilize the resulting material are also considered. In the latter case, the possibilities of solving the problem that inevitably arises in any heterogeneous system, in particular, when a filler is introduced into the polymer matrix, are shown. In addition, surface modification is discussed, as well as the synergistic effect of the use of several co-fillers.

### 3.1. Luminescent Carbon Nanostructures

Composites with carbon nanostructures are usually used to create electroactive polymers [110,111,112], which, in turn, could be used to create electrochemical sensors, such as glucose [113,114,115,116], as well as whole-cell sensors [117,118,119], which make it possible to evaluate such important integral parameters as biochemical oxygen demand based on individual strains [120,121,122] or activated sludge [123,124] or assessment of toxic effects [125]. Such systems can be used, for example, to monitor the fermentation process [126] or toxicant removement [127]. Modern modeling methods show that such materials, depending on the content of the carbon phase, could also be used as antistatic or shielding materials [128].

Equally interesting are the luminescent properties of carbon materials, which make it possible to use them in optical composite sensors. Luminescent carbon nanostructures (CNSs) exhibit a number of the unique properties, including structural features, photoluminescence, and low toxicity. For this reason, they are receiving more and more applications and inevitably growing interest from the scientific community [129]. Naturally, there are also difficulties, including the problems with the separation of the nanostructures mixture after synthesis and the influence of the aggregate state and the environment on their properties [130]. These problems could be solved by the usage of silica matrix. The produced luminescent composite particles exhibit improved optical properties with reduced photoluminescence quenching that leads to a wider application [131]. One can figure out two methods for such material formation: CNSs grafting onto the silica surface and their inclusion into silica particles. Furthermore, the synthesis of multifunctional particles is also available [132]. Such materials combine the fluorescent properties of CNSs with photosensitizing, magnetic and luminescent properties via the addition of titanium dioxide, iron oxide, quantum dots (QDs), and gold nanoclusters (AuNCs) or other functional nanoparticles into the same matrix [129].

Organic room temperature phosphorescent (ORTP) materials represent another important application of carbon nanostructures. They represent an interesting way to develop phosphorescent oxygen sensors with high sensitivity and fast response. Nevertheless, most of the pure ORTP materials are aromatic compound that are stacked into crystals in a face-to-face way. Such tight packing prohibits effective diffusion of O_2_ and hinders sensing application [133]. The switch to a composite system makes it possible to overcome this disadvantage. Polymer (PVA) with layered double hydroxides (LDHs) and carbon dots (CDs) as phosphorescent indicator represents a successful example of ultra-long RTP composite film [102]. The used inorganic matrix has defects on the surface that promote oxygen adsorption. The latter greatly accelerates the phosphorescence quenching and provides linear dependence of the phosphorescence decay intensity to O_2_ concentration. Thus, a synergistic effect is achieved, giving the prospect of developing highly sensitive phosphorescent oxygen sensors.

Another example of the successful application of composite materials is a hybrid structure named as Ru@CD [134]. It represents a covalent binding of Ru(II)-bipyridine complex with carbon dots. The material has a linear calibration dependence (99.1%), good selectivity and stability, while CDs provide a water-compatible supporting matrix.

### 3.2. Enhancement with Metal Nanoparticles

Metal nanoparticles could be used in nano- phostonics and optics for energy transfer, in particular, for the nanoscale localization of the optical field [135] or in the Förster resonance energy transfer (FRET) method [136]. The use of metal nanoparticles plasmon resonance is a promising sensory method. It is based on the fact that the properties of the immobilized nanoparticles change due to the influence of the molecules of the substance (analyte) under study. Several methods have been developed to fix nanoparticles [137]. For example, in situ reduction in gold and silver nanoparticles into chitosan polysaccharide film was reported [105]. The increase in hydrogen sulfide concentration resulted in a decrease in the maximum of the nanoparticles plasmon resonance. The detection limits for the chitosan/gold and chitosan/silver nanocomposites are 5 and 0.1 ppm, respectively.

Another example is the use of melt-processed nanocomposites that represent a combination of the advantages of plasmonic hydrogen detection with polymer technology [138]. The use of colloidal Pd nanoparticles dispersed into the amorphous fluorinated polymer results in nanocomposites that exhibit a high H_2_ diffusion coefficient of 10^−5^ cm^2^ s^−1^ [139]. Despite a thickness of up to 100 μm, such melt-pressed polymer nanocomposites are no longer limited by the diffusion of the analyte to the Pd nanoparticle transducer elements. The response time is as short as 2.5 s at 100 mbar (≡10 vol. %) H_2_. The method is highly scalable and can be used to produce hydrogen sensors en masse. Such cost-effective sensors are critical for the hydrogen economy development.

### 3.3. Nanofibers

Electrospun nanofibers are relatively new materials in optical sensors development, since they have been used in the last 10–15 years. They are quite diverse from a chemical point of view, but at the same time they satisfy one of the main requirements for composite materials—customizability of structure and composition combined with a fairly high versatility [140]. For example, a membrane was made for simultaneous measurement of pH and dissolved O_2_ (DO) [141]. The authors used platinum (II)-5,10,15,20-tetrakis-(2,3,4,5,6-pentafluorophenyl)-porphyrin (PtTFPP) distributed in cellulose acetate (CA) and poly(ε-caprolactone) (PCL) membrane for oxygen sensing. The material was further covered by chitosan coupled with fluorescein 5-isothiocyanate (FITC) in the form of electrospun nanofibers for pH sensing. Thus, it was possible to combine two fundamentally different analytical systems in one membrane due to the blending of nanofibers of a different nature. It is also worth noting that biocompatible materials were used, and the sensor exhibited good response in the required ranges of DO and pH for cells and bacteria incubation monitoring.

Poly(trimethylsilylpropyne) oxygen permeable membrane with incorporated porphyrin dye molecule is another example of successful application of the electrospinning technique [142]. The authors considered several options for modifying the meso-tetrakisphenylporphyrin indicator using the introduction of phenylacetylide substituents on the para position of the phenyl moieties and by switching central metal atom from Pt(II) to Pd(II). Thus, four types of nanofibrous materials were obtained to evaluate the sensing properties towards DO. Phenylacetylide derivatives demonstrated a two-fold enhancement on the lifetime for both types of metal complexes. The effect of silver nanoparticles (the positive effect of which on the properties of optical sensors was shown in the section above) was also evaluated. It turned out that the influence was observed only for Pd complex of phenylacetylide derivative, which exhibits the longest lifetime.

Along with porphyrins, the previously mentioned BODIPYs are also used as dyes in nanofibers. For example, meso-DichlorotriazineEthyl BODIPY (mDTEB) derivative was obtained in order to make it possible labeling of the cellulose nanofibers (CNFs) [143]. The covalent binding and purity of the conjugate was confirmed by the fluorescence lifetime imaging microscopy (FLIM) in a time-correlated single photon counting (TCSPC) mode. The material exhibited excellent photoluminescence properties including high quantum yield and stability over a wide pH range (pH 2 to pH 10). However, it was found that lignin-like impurities reduce the fluorescence of the mDTEB-labeled CNF, via quenching. Thus, it is important to control the CNF production method. No cytotoxicity was observed during in vitro and in vivo assessment. The applicability of the created material for monitoring the respiration of zebrafish (Danio rerio) embryos was confirmed.

### 3.4. Metal–Organic Frameworks (MOF)

Metal−organic frameworks (MOFs) are a class of porous coordination networks with an extremely developed inner surface [144]. They are extremely diverse in physicochemical characteristics such as morphology, porosity and composition and this variability has opened up a large number of possible applications such as energy storage, catalysis, drug delivery [145,146,147]. Moreover, MOFs are also used as template precursors of metal oxides with the desired properties and shape. The encouraging potential for the use of MOF-based matrixes in optical sensors was also presented [148]. MOFs were also used in composite sensors, mainly for indicator distribution due to their highly developed surface [149].

For example, amines sensor was obtained by a combination of Cd(II) 5,10,15,20-tetrakis(4-carboxyphenyl)porphyrin (CdTCPP) and 1,2-bis(4-pyridyl)ethylene (bpe) or 1,2-bis(4-pyridyl)diazene (bpaz) [150]. The structure featured open square 1D channels. Introduction of amine vapours produced significant shifts in the Soret bands of the porphyrin dye used, which made it possible to detect amines calorimetrically. The same structure can be used to detect electron-rich species due to its electron-accepting properties.

The possibility of optical oxygen detection by PCN-224 MOF based on porphyrins and its Pt(II) and Pd(II) derivatives was also investigated [109]. All three materials revealed extremely high oxygen permeability, and the observed bimolecular quenching constants (*k*_q_) were as high as 37,000 (PCN-224), 6700 (Pd(II)PCN-224) and 3900 Pa^−1^ s^−1^ (Pt(II)PCN-224). Such tremendously high values were justified, on the one hand, by large pore sizes, accompanied by the fast gas transport within the internal network, on the other hand, by the spatial isolation of the porphyrin dye in the framework, which prevents self-quenching. The initial PCN-224 structure has fluorescence lifetime of 6.7 ns, which is quenched by a factor of 4.2 with the air. Pt(II) and Pd(II) derivated MOFs possess considerably longer decay times of 18.6 and 390 μs, respectively. Such values made them suitable for trace and ultra-trace oxygen sensing with limits of detection (LOD) of 1 and 0.015 Pa, respectively. Further, the authors demonstrated that obtained sensing crystals could be successfully distributed in different fibrous substrates, such as (poly(acrylonitrile) nanofibers, glass fibres), and flat substrates (TLC silica-gel, poly(amide) filter). It turned out that thermally treated poly(acrylonitrile) and electrospun nanofibers exhibited the same sensitivity as the free crystals. Therefore, such structures are more preferable for practical use. The main disadvantage of the presented materials is cross- sensitivity to humidity. It is reversible when relative humidity is lower than 53% but exhibits a drastic drop in oxygen sensitivity at higher levels. An important role of surface-adsorbed oxygen in the quenching process was confirmed by the decrease in the quenching constant with growing temperature.

Systems of this kind could also be used for other analytes detection, such as nitrobenzene (NB), which should be controlled in wastewater to protect the human health and natural environment. Partial replacement of terephthalic acid (H_2_BDC) ligands of UiO-66 MOF with m-phthalic acid (m-H_2_BDC) followed by modification with Ln^3+^ (Ln^3+^ = Eu^3+^, Tb^3+^) yielded bifunctional Ln@Zr-MOFs [151]. It was found that Eu_10_@Zr-MOFs are suitable for turn-off fluorescent switch of NB, since the latter can easily quench the red emission from Eu^3+^ through a photoinduced electron transfer (PET) process between NB and its ligands. The material exhibits quick response (less than 60 s) and a broad response window (0–180 μM) with *K*_sv_ = 24,459.71 M^−1^. LOD was 1.04 μM and limit of quantification was 3.48 μM. The authors declare strong anti-interference ability and excellent selectivity of the obtained material.

### 3.5. Magnetic Reusable Sensors

Magnetic nanoparticles (NP) are synthetic or natural particles ranging in size from 5 to 100 nm, exhibiting magnetic properties. They are used in many fields of medicine, such as hyperthermia treatment, drug delivery and contrast enhancement in magnetic resonance imaging (MRI) [152]. One of the most important properties of these particles is high recycling ability by means of magnetic separation. For example, composite nanoparticles could be created by the fusion of magnetic spheres into the polymer matrix (PMMA, PS, etc.) along with the oxygen sensing dyes [153]. The produced Fe_3_O_4_@Os1-PS nanoparticles exhibited good dispersibility by optimizing and regulating the number of surfactants, while retaining fluorescence stability. The calibration curve revealed strong linear dependence in the wide range of oxygen concentrations (0–39.30 mg/L), i.e., the resulting material is suitable as an oxygen sensor with high sensitivity. At the same time, the presence of magnetic properties allows one to easily extract the particles by the magnet for further use. Recycled sensing NP kept stable during the operation for one month. Furthermore, the applicability of Fe_3_O_4_@Os1-PS for in vitro metabolic monitoring was also demonstrated using *Escherichia coli* bacteria as an example. Tests have shown non-toxicity and high biocompatibility. Thus, it has been shown that magnetic polystyrene nanoparticles are suitable for oxygen sensing in biological samples.

The combination of porous polyaniline (PANI) and magnetic nanoparticles (Fe_3_O_4_) made it possible to obtain a composite for the adsorption and measurement of gaseous H_2_S [100]. The exposure to H_2_S resulted in a significant shift of the visible spectrum bands (λ_max_ shifted from 577 nm to 616 nm), which allowed optical sensing. The response time at 3 ppm was below 15 min. Thus, the proposed material could find application in hazardous gas detection systems.

Another example of the polymer-based composite sensor is the pH-responsive poly(2-vinylpyridine-co-divinylbenzene) nanogel with covalently attached allyl-PEG capped inorganic nanoparticles (NPs), including magnetic iron oxide (IONPs) [154]. The material was obtained by a simple one-step surfactant-free emulsion polymerization. The late injection of the NPs to the polymerization solution allowed to control their quantity in the conjugate, since at that stage only polymeric radicals were present. It is worth noting that the swelling could be tuned by the variation in the total amount of NPs injected. Moreover, the optical features of the nanogels containing IONPs and magnetic response could also be adjusted.

### 3.6. Polymer Matrix Enhancement

The improvement of the composite material properties could be achieved not only by addition/modification of the filler but also by the alteration of the polymer matrix itself. For example, porosity control can be useful not only to improve the sensitivity and response time of sensors but also can enhance supports for cell scaffolds and biomolecular immobilization [155].

According to the IUPAC classification, porous materials are divided into three classes: macroporous (pore size > 50 nm), mesoporous (pore size = 2–50 nm) and microporous (pore size < 2 nm) materials [156]. The following properties are the main guidelines for researchers in the development of modern matrix materials: high surface area with uniform porosity, the ability to control pore size and their interconnectivity, thermal, chemical and mechanical stability. Due to their long-range order, semi-crystalline polymers are the focus of attention of many scientists. Ultra-high-molecular-weight polyethylene (UHMWPE) is notable among the other commercially available matrixes due to its unique physicochemical characteristics. The recent work by Olga V. Arzhakova et al. presents a comprehensive discussion about the advantages of the UHMWPE mesoporous materials [155]. Different applications have been proposed, including light-weight materials with reduced density, efficient membranes and insulating materials, gas capture and storage systems, porous substrates and scaffolds. The authors have shown that the development of a marked macroscopic porosity could be achieved through the tensile drawing of the UHMWPE samples in the presence of the physically active liquid environments (PALEs), for example, n-heptane, n-decane, isopropanol, etc. The developed process was called environmental crazing and produces nanosized pores smaller than 10 nm. The obtained mesoporous materials have porosity up to ~45%, interconnected pores, high gas permeability (the Gurley number ~300 s) and high-water vapor permeability (1700 g/(m^2^ × day)).

Another way for the tuning of the properties of the polymer matrix is blending of polymers with disparate analyte permeability. For example, the properties of optical oxygen sensors based on polymethyl methacrylate (PMMA), ethyl cellulose (EC), and their blends with different mixing ratios change over a fairly wide range [157]. Such basic parameters of optical sensors as dynamic range and sensitivity were separately increased and decreased as the EC/PMMA ratio decreased in the blended matrix. Dynamic Range (kPa) changed from 0–180 to 0–70, and *K*_SV_ (kPa^−1^) increased from 0.017 to 0.298. The sensing films produced from the mixture with equal content of EC and PMMA exhibited the linear Stern-Volmer plot. The sensitivity tuning approach shown is much simpler and less expensive than the commonly used copolymerization method.

Another example of controlling the properties of a composite by mixing polymers was reported by Duboriz et al. [158]. They showed that polyaniline (PANI)/poly(ethylene terephthalate) (PET) composite films possess an increased surface area. The produced optical sensor gave fast and reversible responses to ammonia or formic acid gas mixtures. In particular, the doped (PANI–HCl)/PET films give linear optical responses to ammonia gas in concentration ranges of 5–200 ppm and 200–920 ppm. The linear response to formic acid was observed for undoped PANI/PET films in the analyte concentration ranges of 15–200 ppm and 200–1600 ppm.

The polymer mixture of polyaniline (PANI)-polyvinyl alcohol (PVA) was used recently to produce the intrinsic optical fiber pH sensor [159]. The absorption properties and refractive index of the PANI-PVA layer are pH dependent, and the exchange of the multimode fiber cladding with the polymer layer produces an optode. The deposed sensing film exhibits porous morphology since the PANI was synthesized in the presence of PVA by means of the reaction in a stable aqueous dispersion. The material exhibited a high sensitivity of 2.79 μW/pH for 2–9 pH range. Evaluation in the analysis of real samples showed good chemical stability and reliability of the sensor compared to the conventional method.

A recent example of the copolymerization-based approach was presented in the work of Shi et al. [160]. The authors used polydimethylsiloxane (PDMS) and isobutyl methacrylate (IBM) to obtain a series of copolymers with different molar ratios. They used atom transfer radical polymerization (ATRP) which gave the narrow polydispersity smaller than 1.21. Oxygen sensors based on the polymer with 50 wt% fraction of PDMS revealed a high-pressure sensitivity of 0.82%/kPa. The observed value is among the highest ones of the reported polymers.

### 3.7. Polymer Composites with Mesoporous SiO_2_

The development of optical sensors is often faced with a situation where the matrix material must satisfy a number of specific requirements that may contradict or interfere with each other. One of the most common is that the material must provide porosity for the transfer of the analyte, but at the same time immobilize the indicator dye [161]. Particularly in the case of a pH sensor, this means that matrix must be hydrophilic to measure in water, while the hydrophobic dye containers must be provided to ensure a high signal intensity. This problem could be solved in different ways, for example, by creating a double-layer composite [162], or by incorporation of polystyrene (PS) nanoparticles (NPs) into the organically modified sol–gel (ORMOSIL) polymer. The latter makes it possible to fulfill all the opposite requirements for the materials of optical sensors. Any hydrophobic fluorophore could be incorporated in this layer, even those one that are susceptible to quenching in water.

The introduction of the mesoporous SiO_2_ phase into the polymer composite also makes it possible to solve the problem of the non-linearity of the calibration dependence. The latter is the result of the inhomogeneity of the indicator dye distribution in the polymer matrix, as well as the presence of crystalline and amorphous regions in it [163,164,165,166].

Due to the developed uniform inner surface of mesoporous materials [156], their use in optical sensors makes it possible to achieve linear calibration [140,162]. However, in some cases, such as measurement in the environment with high biomass content, such materials can facilitate biofouling and degrade response time and performance over time [167]. Fluorine-containing [168], phosphorylcholine [169] and other groups [169] could be incorporated into such materials for anti-fouling protection. However, microvoids still remain accessible for contamination, and exposure to solvents may lead to the indicator dye leaching.

The creation of polymer-based composite makes it possible to combine the advantages of both polymer and mesoporous materials, while eliminating the drawbacks [170,171]. Microparticles of nanostructured SiO_2_ serve as the core for the sensing dye adsorption. The fluorinated polymer matrix prevents the influence of the analyzed environment and serves as the gas permeable medium. Such core-dye-shell structure has linear calibration dependence despite the use of the amorphous polymers. Such a material not only has significantly higher resistance to biofouling compared to traditional polymer sensors such as polystyrene-based [106], but can also be used in rather harsh conditions, for example, for direct measurement in organic media [172]. Not only traditional fluorescent systems could be used as dyes in such materials, but also systems with photoinduced proton transfer, for example [173,174,175].

### 3.8. Surface Modification

The interaction at the liquid-solid interface plays an important role in many processes, including cell interactions, biofouling and fluid transport. They become especially important and noticeable if the particles or surface texture are in the range of colloidal sizes [176]. That is, the modification of macroscopic sensors due to nanosized particles can significantly change their operational properties. Furthermore, not only the adjustment of the sensing material in the bulk is important, but also the surface properties are of no less interest and significance. In this case, composites are a powerful tool for both biofouling control and surface biofunctionalization.

One of the central stages in the creation of a biosensor is the fixation of a biorecognition element. Since it can be different, for example, enzymes, antibodies or even whole cells, the method of fixation also varies significantly. In addition, it is important to take into account the peculiarities of the analyzed sample and analyte when functionalizing the surface. In the latter case, the elimination of steric hindrance for interaction with the sensitive element is of the utmost importance. Two main and most widely used types of bioreceptors can be distinguished among the wide range of known systems: oligonucleotides (single-stranded DNA probes and aptamers) and antibodies. There are many approaches to their immobilization on the sensory surface, each with its own advantages and constraints in terms of flexibility and simplicity, uniformity of the resulting layer, and reliability [177].

Physical adsorption is the simplest method for the sensor functionalization. In this case, receptors are attached directly to the sensor surface due to electrostatic and/or hydrophobic interactions. For example, it was shown that DNA probe with 15-nucleotide sequence with additional *m*-adenine nucleotides at the 5′ end effectively adsorbs on gold [178]. Interestingly, the probe part of the strand turns into an upright conformation, which facilitates further interactions. The spacing between the probes can be regulated in a wide range due to the co-immobilization with the lateral spacer, for example, adenine nucleotides strand of suitable length and relative concentration. It is possible to control the average surface density of the probes in such a way, which in turn controls their hybridization with targets.

The use of chemical crosslinking with the formation of functional monolayers on the sensor surface has the best results in terms of versatility and reliability. This process allows the recognition elements to be firmly attached in a reproducible and controllable manner to the entire surface of the sensor to avoid non-specific adsorption during the operation. For example, this method was used to create an optical sensor for the effective determination of pathological glucose levels [179]. A thin, porous film of chitosan-silica nanocomposite (CSNC) was used as a substrate, on the surface of which Glucose Oxidase enzyme was further fixed. The latter was carried out by the glutaraldehyde chain cross-linking, which led to attachment of enzyme molecules to the surface of the film. The authors evaluated the effect of the surface treatment process on the following important optical parameters: reflectance (R), transmittance (T), surface scattering (SS), internal scattering (IS), and output power (OP). They were all measured simultaneously using a special experimental setup. It was shown that surface functionalization significantly changes all five listed parameters. The highest sensitivity among them for the measurement of glucose concentration in low (<7 mM) and high (>17 mM) ranges was found for SS and IS. The corresponding slopes are 1 mM^−1^ for SS and 1.2 mM^−1^ for IS. The sensor LOD was found to be 0.76 mM, and the quantitative measurement range was 3–30 mM with good reproducibility, selectivity and sensitivity. It is important to note that the created sensors are suitable for single-time use, and this automatically imposes requirements for easy and cheap production method. According to the authors, only 10 mL of CSNC solution is required for the production of 250 samples of sensors, which indicates a sufficient economic efficiency of the fabrication method. It is also worth noting the prospects of the method of multi-parametric sensing, based on image processing, applied by the authors. This direction is an actively growing new area, and in the future, it can make a significant contribution to the development of sophisticated biosensor analysis systems.

Inkjet printing has interesting possibilities for controlled surface modification with functional polymers and hydrogel inks. It may provide a cost effective and scalable method for the local functionalization of biosensors on optical waveguides. For example, a method was shown to obtain a porous hydrogel matrix with high surface densities of the receptor elements [180]. The authors showed that a combination of UV-curable benzophenone dextran (benzo-dextran) and biotin-modified polyethyleneimine (PEI-B) represents an efficient methodology for the detection of biomolecules in a single chip device. A four-channel silicon nitride (Si_3_N_4_) sensor was prepared for the evaluation of the fabrication technology. Mach-Zehnder interferometric (MZI) platform with a waveguide served as the transducer. The results showed that DNA sensing could be done by means of the presented system on the PEI-B surface with a measurement range of 2.5–100 nM and LOD of 0.4 nM.

However, there are situations where the physical or chemical binding of the desired functional groups or molecules to the surface of the sensor is difficult, for example, when dealing with fluorinated polymers [165]. In this case, particles or fibers with desired properties could be physically embedded into the surface of the material [181,182]. One can consider, for example, modified detonation nanodiamonds (DND) as such a platform with tunable and controllable properties. It was demonstrated that DND formed a strong complexes with human serum albumin (HSA), while not affecting platelet aggregation and exhibiting weak antiradical activity [183]. The interaction occurs in subdomain IB (digitoxin, K_b_ = 20.0 ± 2.4 L·g^−1^) and in subdomain IIA (warfarin, K_b_ = 3.7 ± 0.1 L·g^−1^). The observed binding resulted in inhibition of the HAS esterase activity. A genotoxic effect was revealed towards PBMCs by dispersions of DND with *C* = 0.0012–0.15 wt%, which, however, did not affect HEK293 cell line during the cellular proliferation experiment. In the latter case, no cytotoxic effect was observed up to 0.01 wt%.

DND could be modified/functionalized to tune their properties for further incorporation into polymer matrices. For example, a method was reported to improve the properties of the proton conducting membranes [184]. The authors used the Aquivion^®^ type perfluorinated copolymer with short side chains and doped it with DND. The introduction of the ionogenic groups into the structure on the DND surface allowed to tune the proton conductivity of the fabricated composite membranes. The properties of the obtained material significantly overcome the Nation^®^ type membranes with long-chain and that makes them promising for use in the hydrogen fuel cells.

Modified nanodiamonds could be used to control the wetting and biofouling properties of the surface. It was shown in the example of polymer-based optical oxygen sensor [185,186]. Both quantity of nanodiamonds and their functionalization effect on the properties of the modified surface. It was shown that the same fluorinated polymer after treatment can acquire bactericidal properties or vice versa good biomaterial adhesion. This opportunity was reached after the optimization of the anchoring conditions, which allowed realizing the accurate control of wetting and biofouling properties. Both in vitro and in vivo experiments showed that sensor surface with fixed amine modified DND demonstrated the inhibition of biological activity. However, a large number of DNDs with mixed modification (amide and acid chloride groups on the same particle) resulted in good adhesion of the biomaterial since the presence of polar groups enhances the wetting. The latter could be used to create a biosensor on-site [107]. It was shown that cell adhesion produced a stable biosensor, which responded glucose injections. The electrostatic interaction of the cell wall with the anchored polar groups was responsible for the fixation during the first 5 days of incubation. Polysaccharide matrix contribution became significant only on the 7th day, which resulted in noticeably superior response to substrate additions due to the greater number of the attached cells. However, 1.5 h of incubation was sufficient to form a biosensor with reproducible pronounced response. Thus, the presented optical sensor with DND-modified surface allows one to quickly isolate the microbiome and the prepared bioreceptor could be used further for the substrate specificity studies or for the assessment of the toxic influence resistance.

In the case of DND, the above requirements for the uniformity of the distribution of functional groups on the modified surface require the development of experimental techniques that stabilize the liquid dispersion and prevent premature deposition. For example, nonionic surfactant Igepal CA-630 could be used to stabilize the water and DMSO dispersion of DND [187]. It remains stable for at least 7 days, while the dispersions in DMSO retain their aggregation resistance for 3 days. This allows one to evenly distribute the particles over the surface of the material being treated.

Materials with tunable and reversible dry adhesion adjusted rapidly by the magnetic field were also reported [188]. The composite material was obtained by the combination of the core made of a magnetorheological polymer gel (MRPG) with a cylindrical polydimethylsiloxane (PDMS) shell. The obtained pads are susceptible to the influence of the magnetic field, which may form the interface stress responsible for the tuning of the surface adhesion. It was shown that the larger adhesion could be achieved when the edge stress is the lowest. The application of 5 A current resulted in the change of the pull-off force from 0.442 ± 0.008 N to 0.505 ± 0.007 N.

### 3.9. Improving the Mutual Adhesion of the Polymer Matrix and Filler

Since a composite is a material consisting of at least two phases separated by an interface border, it is essential that its components have appropriate adhesion to each other [189]. Otherwise, a significant decrease in mechanical strength could be observed, which may result in rapid destruction of the heterogenous material [188,190,191,192,193].

If metal inserts are used in the composite, their surface texture changes can be made through a combination of mechanical and chemical processing in order to increase adhesion at the metal-polymer interface and provide a high bearing capacity with adhesive joint [189]. The corundum mechanical treatment followed by the proper chemical etching gave the best results. The adhesion force value increased almost a hundredfold compared to the unmodified samples.

The influence of abrading treatments on carbon fiber/cyanate ester were also investigated [190]. The surface tension-based measurements showed that surface hydrophilicity and morphology could be, to a large extent, dependent on the coarsening treatments. Thus, it was shown that mechanical processing of the filler affects surface morphology, adhesion, and roughness. All this affects the properties of the resulting composite. For glass fiber (GF)/polyester composites, it has been shown that plasma treatment makes it possible to control the mutual adhesion and shear properties of the material [191].

However, fluorinated polymers hold a special place among many materials used as polymer matrices [194]. These materials exhibit exceptional properties in terms of chemical inertness, durability, and photodegradation resistance [195,196,197,198,199]. Because of the low surface energy, fluoropolymers have extremely poor adhesion to almost all materials, that makes it difficult to use them as composite matrices [200,201]. Fluorinated silanes could be used to improve the mutual adhesion of components in such systems; however, in a recent work by Krapivko et al. showed that this approach may not be sufficient for perfluorinated materials such as polyhexafluoropropylene (PHFP) [202]. The authors proposed the principle of structural coherence that increases mutual adhesion of the polymer chain to the specially designed fluorinated silane structure. The surface modification was combined with a well-known aluminum foil anodizing technique [203,204,205]. The produced composite material was proved to be stable and promising for use in harsh environments.

### 3.10. Hybrid Materials with Several Co-Fillers

Composite materials allow combining several co-fillers, which represent different phases, to achieve the maximum synergistic effect. As shown above, the localized surface plasmon resonance (LSPR) induced by the metal nanoparticles (NP) could greatly enhance the intensity of phosphorescence. This phenomenon is largely dependent on the overlap of the noble metal absorption peak with the absorption peak of phosphorescent dyes. The largest effect is achieved when there is maximum overlap.

Yin et al. showed an example of such a synergistic effect in improving the performance of an optical molecular oxygen sensor [108]. The authors took as a basis a composite sensor consisting of mesoporous silica gel particles with an adsorbed dye PtTFPP, which are distributed in a polymer matrix [170,171]. The tunable plasmonic resonance was demonstrated by means of SiO_2_/AuNPs obtained by seeded growth method. It was shown that the sensitivity of the sensor is strongly dependent on the enhancement of phosphorescence in a wide oxygen concentration range (0–21%). The largest enhancement of phosphorescence with a factor of 7 was achieved by varying the NP size to adjust their absorption spectrum to that of the PtTFPP indicator dye used. Further improvement of the sensing system was done by the optimization of the NP content to eliminate self-quenching and the nonradiative energy-transfer (NRET).

Another obvious advantage of composite materials is the combination of several analytical systems in a single sensor coating. Different sensing particles containing appropriate molecular probes (for example, for O_2_, CO_2_, pH or temperature measurement) could be fused into the host polymer matrix. Moreover, the obtained sensor cocktail may be produced in a sprayable form for ease of use in imaging and optical sensing [206]. The prepared solution could be applied (sprayed) at low temperature to the surface of interests. The stable film is obtained by the gelation of the composition due to the temperature growth. The described procedure yields a tightly adhered sensing layer. The main requirement for the creation of such materials is the spectral separation of the luminescent signals of the indicators used, otherwise there will inevitably be a cross-talk of signals, which complicates the measurement [207].

In addition, the creation of a polymer nanocomposite can make it possible to combine molecules of different nature in one material. For example, it was shown that core-shell structured poly(styrene- block-vinylpyrrolidone) nanoparticles with average diameter 180 nm could incorporate lipophilic indicator dyes to produce sensor layer capable of simultaneous measurement of oxygen and pH [208]. Oxygen sensitivity was achieved by the entrapment of the Pt(II) meso-tetra(4-fluorophenyl)tetrabenzoporphyrin (PtTPTBPF) dye into the polystyrene core. pH measurement was provided by BF_2_-chelated tetraarylazadipyrromethene dye (aza-BODIPY) incorporated into the polyvinylpyrrolidone shell. Light harvesting system allowed to increase the brightness of the pH sensing units by more than 3 times. The obtained sensing layer exhibited good resolution: 0.5–2.0 hPa (approx. 0.02–0.08 mg/L) for oxygen concentrations below 50 hPa and 4–8 hPa (approx. 0.16–0.32 mg/L) at ambient air concentrations (approx. 200 hPa). The achieved pH resolution was 0.03–0.1 pH units with p*K*_a_ 7.23 ± 1.0. The created sensor was tested during continuous monitoring of the enzymatic conversion of Penicillin G to 6-aminopenicillanic acid catalysed by Penicillin G acylase.

## 4. Optical Polymer-Based Sensors in Environmental Objects and Biological Systems

This section considers relevant practical examples of the use of the reviewed technologies for creating optical sensors. Undoubtedly, the most demanded is the measurement of physiologically important analytes such as O_2_, CO_2_, pH in biological systems, environmental and industrial objects. However, the range of possible analytes is not limited to these three substances, and such recent examples are given in the section.

### 4.1. Measurent of O_2_, pH and CO_2_

#### 4.1.1. Food Packaging

The modern food industry is an actively developing area, in particular, intensive progress is underway in the field of new food packaging technologies. The main efforts are aimed not only at the introduction of new areas of knowledge (such as biotechnology or nanotechnology), but also take into account the environment awareness. An important growing trend is the development of “smart” packaging that can control the safety and quality of products, extend food shelf-life, as well as taking care of the environment. Their implementation requires, on the one hand, changes in production technologies, but on the other hand, it can change the practice of selling and the lifestyle of consumers [209].

One of these technologies measures oxygen gas concentrations inside food packaging [210]. Unlike other methods, the measurement does not require opening the package. It is enough to place a small sensitive foil into it at the production stage. Of course, the sensing material must be inert to the main product [211], and the use of composites described in the previous section facilitates this task. It is important that for practical measurements in a store, the user can even use a smartphone with the appropriate software. In this case, the flash of the smartphone, together with an optical filter, is used to excite the luminescent membrane, and the rear camera is used to register the analytical signal [212]. The presence of oxygen in the analyzed medium leads to quenching of the luminescence. Representation of an analytical signal as a ratio is the most convenient in terms of stability and accuracy of measured readings. In this case, the measured intensities are compared in two different wavelength ranges corresponding to the emission and absorption peaks of the sensitive membrane or the emission of the oxygen-sensitive and reference dyes. It is important to note that the thermal dependence of modern sensor materials is significantly reduced, as is the cost of their production [213,214]. The described approach shows that modern sensory materials can be easily integrated into smart packaging, and the current state of the pocket electronics makes it possible to avoid the need for a special measuring equipment.

#### 4.1.2. Microparticles-Based Ink

This technology is a simple and inexpensive way to fabricate sensor arrays suitable for both point-to-point measurements and oxygen distribution mapping. For example, the possibility to print multiple sensor patches at ones was demonstrated [215]. The authors used a polyvinylidene chloride film as a substrate and ink mixture prepared using the monodisperse polystyrene microparticles with immobilized oxygen-sensitive PtTFPP dye. A 50% mixture of ethanol and water with suspended high molecular weight polyvinylpyrrolidone was used as a dispersion medium for an ink mixture. The polymer used provides enhanced adhesion and compatibility with the most common polymer substrates. The obtained patches exhibited reproducible and fast response in a wide range of oxygen concentrations (0–21%) with linear Stern-Volmer plot (R^2^ > 0.99) and sufficient sensitivity (I_0_/I_21%_ > 1.55). It was found that the intensity of the printed material luminescence mainly depends on the concentration of the fluorophore and, to a lesser extent, on the size of the polystyrene particles. It was shown that patches are suitable for measurement with both a multi-frequency phase fluorometer and a smartphone. The good uniformity of the resulting printed surfaces made it possible to carry out two-dimensional measurements with mapping of the oxygen distribution.

Non-fluorescent color indicators can also be used for ink-printed sensor production. For example, a quantitative colorimetric oxygen sensor was produced using the conversion of bisphenalenyls (PQPLs) into aromatic endoperoxides (EPOs) as the indicator [216]. One of the key features of the PQPLs is the ability to generate singlet oxygen, i.e., they possess self-sensitizing reactivity and do not require an external photosensitizer. The rate of photooxygenation depends on the electron-donating ability of PQPLs substituents, and it allows an easy way to tune the sensitivity. The produced EPOs are stable under ambient conditions but could be converted back to PQPLs by the thermal action. The produced sensing films exhibit visible to the naked eye rapid red-to-colorless transition in the presence of oxygen with a very low LOD of <5 ppm O_2_. The reproducibility of the characteristics for the material was shown.

#### 4.1.3. Cementitious Materials

Cementitious media are one of the examples where the introduction of optical methods for pH and O_2_ monitoring allowed measurements to be taken at a qualitatively new level [217,218]. The key benefit of optical methods in comparison with “wet” chemistry traditionally used in this field is that they allow continuous, non-contact measurements at intervals down to seconds. For example, pH change was monitored in calcium aluminate, calcium sulfoaluminate and OPC/slag cements [217]. It turned out that the measured dependence correlated with the heat of hydration. Thus, it is possible to monitor the formation or aging of solid phases in situ in a non-destructive way. Highly reproducible data obtained for hydration and consolidation reactions in highly alkaline cementing systems, for example, pastes, slurries, and hardening materials, opens up new possibilities for experimental investigation.

Optical sensors can help in solving another important problem—the search for leaks from cement structures that perform retaining functions. Such leakages can occur when cracks appear in the cementitious material, and they may have devastating environmental consequences. The use of self-healing concretes has been proposed as a solution. The principle of operation of such materials is based on the addition of bacteria during manufacture, capable of inducing the precipitation of CaCO_3_, which in turn leads to healing of the resulting cracks even before leakage occurs [218]. The key thing in the development of such materials is the biocompatibility of the host material with the biological object, and the pH of the medium plays a decisive role. This was confirmed practically in the study of biological activity in cement by introducing superabsorbent polymers with encapsulated endospores of *Bacillus alkalinitrilicus* into cracks. Measurements of the respire activity were made using oxygen optodes. It was shown that lowering of the pH of the cement from >11 to <10 by incorporating fly ash, as well as increasing the hydration time, resulted in a significant increase in bacterial activity.

#### 4.1.4. Marine Environments

One of the requests in oceanography is the ability to measure and map the content of various substances, primarily associated with physiological processes, such as dissolved O_2_, CO_2_ and pH [219]. The sensors are used both for continuous in situ observation using various autonomous platforms, as well as for profiling using underwater vehicles and ships.

Optical methods in the described tasks have a number of advantages. For example, a unified optoelectronic platform can be developed that is customizable for the measurement of a specific analyte by replacing the excitation source and/or light filters [220,221]. This approach significantly reduces development and maintenance costs. Practical tests were performed for the developed platform, which differed in the conditions for performing measurements. They differed in the duration of the measurements: long-term monitoring from 5 days to 8 weeks and profiling (several hours); environmental conditions were also varied, including temperature 9–25 °C and salinity: 6–33 PSS. Both stationary variants and fastening on mobile vehicles have been tested. Practical experiments have clearly shown the critical importance of choosing the right calibration strategy, otherwise systematic distortion of the results can occur, which is unacceptable, especially for long-term measurements. The importance of choosing an anti-biofouling strategy to extend measurements in high biomass environments has also been demonstrated.

Another interesting example of the use of optical sensors is the integration of monitoring systems and vehicles capable of detecting and mapping CO_2_ leaks from offshore carbon capture and storage (CCS) sites [222]. The evaluation of the effectiveness of such a vault requires the development of a reliable and cost-effective control system since the CO_2_ storage is considered as one of the main ways at the current stage of the fight against climate change. A method has been demonstrated to create a plume model based on spatial pH distribution data. They were obtained using a remotely controlled underwater vehicle with a spatial orientation system and a fixed optical sensor.

It is important to note that, for the simultaneous measurement of the parameters in the examples given, separate sensors are currently used, which complicates and increases the cost of the design. As shown in the previous section, the combination of different sensor materials in one foil is possible and is achievable only when using composite materials. The main requirement in this case is non-overlapping fluorescence spectra of the indicators used. For example, simultaneous detection of oxygen and carbon dioxide was reported [223]. Simultaneous detection was provided by the combination of well-known PtTFPP as the O_2_-sensitive dye, phenol red combined with CdSe/ZnS quantum dots as the CO_2_-sensitive dye, and unmodified CdSe/ZnS QDs as the reference signal. Poly(isobutyl methacrylate) (PolyIBM) was used as the host matrix for the immobilization. All dyes used are capable of absorption in the near UV region, so a 380 nm LED was used for excitation. An increase in the O_2_ concentration leads to quenching of the fluorescence of the Pt(II) complex, and the appearance of CO_2_ leads to an increase in the QDs fluorescence intensity. The response and recovery times were 10 s/35 s for O_2_, and 20 s/60 s for CO_2_, respectively. The sensitivities of the ratiometric dual sensor were approximately 13 for O_2_ and 144 for CO_2_, respectively.

#### 4.1.5. High Pressure Measurement

The ability to perform measurements under high hydrostatic pressure is one of the clear advantages of optical sensors over electrochemical ones, such as pH electrodes. However, such harsh conditions can significantly change the properties of the analytical system, and an assessment of such an impact on various types of sensory materials has been carried out recently [224]. The authors immobilized commonly used indicators into porous and nonporous matrix materials to evaluate the influence of the structure on the observed dependencies. A custom chamber was made that allowed cyclic pressure stepwise change up to 200 bar. The authors used Pt(II) benzoporphyrin and Ru(II) polypyridyl complexes as indicators. They were immobilized in different microparticles, such as crosslinked polystyrene, poly(phenylsilsesquioxane), silica gels of different porosities, ZIF-8 and UiO-66 MOFs. Composite sensor was obtained by distribution of the microparticles with dyes into gas permeable Hyflon AD and silicone matrices. A comparison was made with homogeneous polystyrene and hydrogel films and nanospheres of non-porous polystyrene directly dispersed in water. All of the listed materials proved to be stable at high hydrostatic pressure, but their response to change was different. Sensors with porous materials showed a decrease in the observed concentration of oxygen, although the actual content did not change. The difference was from −0.02 to −0.45 mg O_2_ L^−1^ H_2_O per 100 bar. Kinetic experiments with high temporal resolution showed that during the first seconds after the change in pressure, spikes were observed due to the redistribution of oxygen between two solid phases of the composite. The changes were fully reversible. The “positive” or “negative” direction of the spike in the dependence of the observed concentration depends on which of the materials is more ductile—the porous particles with the dye or the host polymer.

#### 4.1.6. Plant Roots System

An aeration system is necessary for root growth and plant survival, which is an important problem in flooded and swampy soils [225]. Special barriers are formed by many wetland species to restrict radial O_2_ loss (ROL) from roots to the rhizosphere. Such structures significantly enhance the diffusion of O_2_ in the longitudinal direction, i.e., from basal parts towards the root tip. It also prevents the entry of phytotoxic compounds into the root. However, ROL from roots provides an essential source of oxygen for the oxidation of toxic compounds in rhizosphere [226].

There are commercially available systems with planar optode suitable for the direct measurement of O_2_, CO_2_, and pH in rhizosphere of wetland plants [227]. The setup consists of a water aquarium with a fixed plant root and a sensing foil applied to the top. They are isolated from external illumination using a special box. A software-controlled LED is used to excite the luminescence of the indicator and the data is collected by a special camera in the form of images enabling mapping of the spatial distribution of oxygen.

### 4.2. Other Analytes

#### 4.2.1. Uric Acid

Optical detection of uric acid in solutions was demonstrated by means of hybrid nanomaterials consisting of 5,10,15,20-tetrakis(4-amino-phenyl)-porphyrin (TAmPP) doped with copper nanoparticles (CuNPs), platinum nanoparticles (PtNPs), or both types (Pt@CuNPs) [228]. The addition of nanoparticles, as in the case of other luminescent sensors, made it possible to improve the range of measurable concentrations. UV-Vis spectrophotometry was used for detection, and the morphology was analyzed by means of atomic force microscopy (AFM). The most stable response with the highest sensitivity was demonstrated by a composite with porphyrin and PtNPs. The uric acid detection range was found to be 6.20 × 10^−6^–1.58 × 10^−5^ M, and the substances present in the human environment did not affect the measurement even in a very high concentration.

#### 4.2.2. Hypochlorous Acid/Hypochorite

Combination of sodium alginate (SA) and RhB-AC placed into the interpenetrating polymer network (IPN) hydrogels allowed to obtain a biocompatible material for HClO/ClO^−^ determination [229]. This material is suitable for use as smart wound dressings or as a drug scaffold with controlled drug release. The in vitro addition of hypochlorous acid resulted in a significant change in the fluorescence of the hydrogel, confirming its ability to detect the analyte. Good biocompatibility of the material was confirmed by cell culture and toxicity tests. Both the hydrogel drug scaffold and the material itself exhibited excellent healing effects when they were used for wound healing in vivo.

Another system for the determination of HClO/ClO^−^ was demonstrated using a novel near-infrared-emitting aza-BODIPY derivative [230]. The fluorescent probe with two tellurium atoms at two upper benzyl rings showed fluorescent “turn-on” effect at 738 nm and high selectivity [230]. The photoinduced electron transfer caused by the presence of two tellurium atoms leads to significant quenching of the fluorescence of this probe. However, the exposure to HClO/ClO^−^ resulted in the oxidation of both electron-rich tellurium atoms, and a strong fluorescence emission was observed at 738 nm with the quantum yield of 0.11. Calibration plot was linear in HClO/ClO^−^-concentration range of 0–30 μM; LOD was 0.09 μM in acetonitrile aqueous solution. Ability to work over a wide pH range (from 2 to 10) should also be mentioned. Applicability for endogenous and exogenous detection and imaging of HClO/ClO^−^ in living cells was confirmed by the experiments in RAW264.7 cells.

#### 4.2.3. Ammonia

The possibility of performing a simultaneous measurement of gaseous O_2_ and NH_3_ was demonstrated using a sensor in the form of cellulose fibers impregnated on one side with the PtTFPP dye, and on the other with eosin-Y [231]. Both indicators were initially distributed in mesoporous sol-gel matrix, which was further applied to the support fibers. The concentration of each gas is determined by the quenching of the fluorescence intensity of the corresponding dye. Each gas, ideally, only affects one of the indicators. However, the synchronized measurement of several analytes using the same sensor material often suffers from cross-sensitivity effects. In the case of the created sensor, it turned out that the fluorescent peak associated with the O_2_ measurement is also quenched by NH_3_ and vice versa. The authors proposed a new analysis method to eliminate such interference. They systematically studied the mutual influence of analytes on the recorded signal and proposed an improved algorithm for calculating the true concentration, taking into account the observed cross-sensitivity. The proposed version of the data processing made it possible to reduce the error of oxygen determination from −11.4% ± 34.3% to 2.0% ± 10.2% in a complex media.

#### 4.2.4. 1-Anthraquinonsulfonic Acid

1-anthraquinonsulfonic acid (AQ) is an important marker in medical investigations of congestive and cancer diseases, pancreatic fibrosis, microbial tests and in diabetes. Possibility of measurement was demonstrated by means of the porphyrin-based composite material [232]. The authors synthesized new 5,15-bis-(3-hydroxyphenyl)-10,20-bis(3-methoxyphenyl)-porphyrin (trans-A_2_B_2_-porphyrin) and further received its Pt(II) derivative, namely Pt-trans-A_2_B_2_-porphyrin. The indicator molecule was linked to gold nanoparticles (AuNPs) to improve optical properties (see Section 3.2 for an explanation). The obtained material showed the possibility of measuring AQ in the concentration range from 2.419 × 10^−8^ M to 2.5 × 10^−7^ M which corresponds to the desired physiological range. An assessment of the crosstalk effect was performed, since the analyzed physiological samples have a complex composition, which may be the source of the interfering factors. It turned out that there is no strong influence on the AQ measurement process, even at a 50-fold excess of the impurity concentration relative to the target analyte.

#### 4.2.5. Manganese Ions

The development of multifunctional materials capable of monitoring toxic metal ions in water is a significant task, necessary to maintain a clean and sustainable environment. To control the content of manganese ions, the new carboxyl-substituted A_3_B porphyrin, 5-(4-carboxy-phenyl)-10,15,20-tris-(4-methyl-phenyl)–porphyrin, was synthesized and a novel composite material was created [233]. The functionality toward Mn^2+^ detection from polluted waters and from medical samples was evaluated. The plasmonic porphyrin-k-carrageenan-AuNPs material detected Mn^2+^ in the concentration range from 4.56 × 10^−5^ M to 9.39 × 10^−5^ M (5–11 mg/L). Such concentrations may be useful for monitoring the health of people who have been exposed to contaminated water sources or who have consumed large amounts of manganese in their diet.

#### 4.2.6. Hydrogen Peroxide

Continuous precise detection of hydrogen peroxide (H_2_O_2_) at low concentrations is essential for clinical, pharmaceutical, biological, and environmental analysis. One of the most convenient and adaptable methods for a specific application is optical detection using probes and nanomaterials [234]. An example of such composite material with fast and reversible response has been reported recently [235]. The fibrous silica particles (KCC-1) were used for in situ growth of the platinum nanoparticles (PtNPs) inside their pores at the first stage of the sensor fabrication. The obtained nanocomposite was further embedded into a hydrogel matrix with an oxygen sensing dye PtTFPP. The measurement principle is as follows: hydrogen peroxide is catalytically converted into molecular oxygen by immobilized PtNPs, the latter being determined by the optical sensor based on phosphorescence quenching. The sensor demonstrates a fast response (less than a minute), which is completely reversible, due to the use of a highly porous KCC-1 structure, that imparts good permeability and at the same time allows creating a high local concentration of nanoparticles. Thus, the advantage of composite structures in optical sensors was practically demonstrated. The measurement could be done in a wide range from 1.0 µM to 10.0 mM, while the sensor requires only 200 µL of sample for analysis. Thus, the presented sensor, due to the described advantages, can meet all industry requirements for real-time measurement and fill a vacancy in the market.

### 4.3. Biomolecular Applications

Optical sensors allow continuous monitoring of various biochemical substances. The resulting data provide insight into physiology and health. However, there are known difficulties in the development of sensors for such measurements, associated with the need to achieve biocompatibility, non-toxicity and compatibility with aqueous media, since indicators are often insoluble in water. For these reasons, silk protein-based biomaterials are particularly useful as a scaffold material for optical sensors. They possess an exceptional amphiphilic chemistry that leads to the stabilization of protein and indicator dye in an aquatic environment. A practical example of the implementation of this approach was demonstrated using an optical oxygen sensor [236]. The authors used silk films with water-insoluble dye Pd(II)tetramethacrylated benzoporphyrin (PdBMAP) and tested this material in vitro and in vivo. The stabilization of the composite was observed due to the self-assembling of physically cross-linked protein network. A twofold quenching of the lifetime of excited state of the dye was observed at a dissolved oxygen content of ≈31 μM, which suggests the applicability of the material in the physiological range of concentrations. In vitro test has shown cytocompatibility of the material, while in vivo implantation in rats confirmed the biocompatibility and displayed those mechanical properties are suitable for subcutaneous implantation. In addition, the applicability of the sensor material to real-time measurements under various physiological conditions (normoxia, hyperoxia and hypoxia) was shown.

Another approach to the development of a targeted measurement system is the chemical modification of an indicator molecule in order to give it the ability to bind to a specific target or to penetrate the lipid membrane in a biological sample under study. For example, meso-substituted BODIPY with the butanoic acid residue was covalently binded to the thioterpene moiety was performed to examine the membranotropic effect to erythrocytes and to evaluate the practical application of the conjugate in bioimaging [237]. The obtained dye exhibited high fluorescence quantum yield at 514–519 nm and high photostability. Moreover, the absence of erythrotoxicity was confirmed despite the fact that the indicator effectively penetrates the membrane of erythrocyte. Thus, it can be concluded that conjugation of the hydrophobic dyes with a thiotherpenoid is the right way to impart the affinity to biostructures, e.g., blood components.

Biocatalytic processes represent another field where optical sensors are indispensable and can provide unique information. O_2_-dependent biocatalytic reactions could be mentioned an example. If the process takes place in a bulk liquid, then the supply of the required amount of oxygen can be simply organized by aeration with air. However, customized O_2_ supply solutions are required when biocatalysis occurs in a spatially limited microstructure of the solid support. Release of O_2_ through controlled decomposition of hydrogen peroxide is considered as one of the most promising options, however this requires a means for continuous spatiotemporal measurement. An example of such a system, which uses optical sensing of soluble O_2_ formed from H_2_O_2_ in a porous carrier by immobilized catalase, was shown recently [238]. The obtained O_2_ was consumed by the oxidation reaction of D-methionine that was co-immobilized in the same carrier with the catalase. The optical sensor made it possible to determine that the reaction rate exhibits linear dependence on the internal O_2_ concentration up to the level of the air saturated solution. In addition, it was practically shown that such an oxygen generation system makes it possible to achieve a 1.5-fold acceleration of the reaction compared to air aeration. Taken together, these results show how the unique spatial measurement capabilities provided by optical sensors can be used to develop a method for the controlled delivery of O_2_ from H_2_O_2_ to enzymes immobilized in a carrier. Such an integrated strategy will be especially useful in biomolecular engineering to overcome the limitations of O_2_ supply through gas-liquid transfer.

### 4.4. Organ-On-Chip, Lab-On-Chip and Microfluidics

Compact organ-on-chip systems represent encouraging methods for in vitro research in biology, medicine and pharmaceuticals. Their main advantage over conventional cell culture platforms is the improved resemblance of the culture environment [239]. The ability to control both the biological responses of the cultured cells and the cell culture conditions is the most important aspect of these systems. On-chip integration of in situ analysis methods provides improved temporal resolution, faster readouts and continuous measurements of such properties as the metabolic activity, the release of particular molecules, rate of proliferation and differentiation [240]. Therefore, the developed models of organs and diseases could provide more information. Many sensor types have been integrated into lab-on-a-chip systems for chemical analysis, including electrical, electrochemical and optical sensors. Recently, some of these measurements and monitoring principles have also been applied to organ-on-a-chip systems.

Successful integration of a sensor into a microfluidic system requires compatibility with the fabrication process. At the same time, in the case of optical sensors, it is necessary to take into account such specific features as ensuring long-term stability despite the sensor degradation, biological fouling, and peculiarities of manufacturing processes [241]. For example, a manufacturing technology for an optical sensor of dissolved oxygen based on platinum octaethylporphyrin (PtOEP) immobilized in a polydimethylsiloxane (PDMS) membrane was presented [242]. The choice of matrix was justified by the fact that microfluidic devices are usually made from PDMS, and therefore the authors investigated the in-detail features of the fabrication process using standard microfluidic materials and technologies. The outstanding chemical and mechanical properties of PDMS, such as anti-biofouling characteristics and high oxygen permeability, made it possible to achieve sensors with better sensitivity compared to other matrix materials. The achieved detection range was from 0.5% up to 20% in a gas media, and from 0.5 mg/L up to 3.3 mg/L in liquid media at 1 atm, 25 °C.

In addition to optical fluorescent sensors, chemiluminescent (CL) systems also take an important place in microfluidic systems. For example, chemiluminescence (CL) of luminol catalyzed by AuNPs could represent an easily implemented alternative to enzyme-based methods. The aggregation of NPs leads to significantly enhanced CL signals, therefore substances disturbing such a process will lead to weakening of the signal that could be easily detected. An example of the use of such a concept was demonstrated in the detection of sulfadimethoxine (SDM) using the CL microfluidic flow-injection platform with homogeneous aptamer-based assay measurement [240]. The most important for the successful implementation of the method is the efficient mixing of the components, i.e., AuNPs, aptamers, and analyte. Two-dimensional (2D) and three-dimensional (3D) mixer designs were examined. It turned out that the first one could not provide sufficient mixing and a laminated 3D 5-layer microfluidic mixer was developed. The device has been optimized to not only improve the quality of mixing but also to ensure the image acquisition by a CCD camera. The comparison of luminol with its derivative m-carboxy luminol, which is more hydrophilic, showed that the latter exhibits a tenfold increase in the signal and more reliable readouts. The system demonstrates an extraordinary range of detectable concentrations of SDM: 0.01–1000 ng/mL (5 orders of magnitude), and LOD of 4 pg/mL. These impressive results show not only the superiority of the method in determining SDM but also that the underlying method can be applied to other analytical tasks in the food industry and in environmental control.

Lab-on-chip systems could also be used in the analysis of environmental objects. For example, they are capable to perform simultaneous measurements of water chemistry parameters with high accuracy [243]. At the same time, they can be used even in extremal conditions, for example, at depths down to 6000 m. The latter system is produced under license by Clearwater Sensors Ltd. (Southampton, UK) and utilizes the in situ lab-on-chip technology from the University of Southampton and the National Oceanography Centre (NOC). More than 200 successful cases are known, including analysis at a depth of about 4800 m, in muddy estuaries and rivers, as well as for up to a year in seasonally ice-covered regions of the Arctic. A set of methods is used combining optics with microfluidics and electromechanical valves and pumps which allowed to mix water samples with reagents and detect the response. Chemical and biological parameters could be monitored simultaneously, including Nitrate, Nitrite, Phosphate, Silicate, Iron, and pH.

### 4.5. Imaging

One of the unique features provided by optical sensors is the ability to obtain the spatial distribution of the analyte concentration, i.e., mapping. Such information makes it possible to establish, for example, the structure of biofilms, as well as to monitor their metabolic processes in dynamics [244]. All this requires tools with the necessary measurement sensitivity and resolution to detect these changes, which were lacking until recently. An example of the possibilities offered by optical composite sensors are nanosensors that include pH-sensitive fluorophores covalently encapsulated with a reference pH-insensitive dye in an inert matrix of polyacrylamide nanoparticles [245]. They can be used for real time monitoring of three-dimensional changes in pH for biofilm formers. The potential application of nanosensors for detection of sugar metabolism in real time gives a chance to improve oral health through the therapeutic solutions.

Another important area of imaging application is the study of tissue oxygenation, which plays an important role in tumor development and treatment [246]. Optical methods for measuring, for example, oxygen using phosphorescence quenching have been known for a long time, and there are a number of commercially available systems, including those for planar measurements. However, in the case of bioimaging in live systems, the creation of biocompatible phosphorescent complexes is still a difficult task [247]. As an example of a molecule suitable for the described requirements, one can mention meso-tetra(sulfophenyl)tetrabenzoporphyrin Pd(II) (TBP) [248]. In vivo experiments with S37 tumor showed that oxygen level in tumors was lower compared to normal tissues where TBP phosphorescence was completely quenched. Photodynamic therapy in the solid tumor and in the muscle resulted in the increase in TBP phosphorescence lifetimes that confirms oxygen consumption during treatment and probably the blood flow stops.

Spherical microprobes with phosphorescent dyes are well suited for the tissue engineering to monitor the oxygen concentration in close proximity to the cells during their growth phase. A fluorescence microscope could be involved for excitation and image acquisition when the standard seed trays like glass dishes are used [249]. However, the imaging of large biological samples by means of such laser scanning fluorescence lifetime imaging (FLIM) is practically unrealizable due to the small field of view (typically less than 1 mm). However, a system was also shown that allows FLIM to be performed on macroscopic objects up to 18 mm in size, with a lateral resolution of 15 μm [250].

Oxygen mapping in a variety of biological milieu could also be performed by boron nanoparticles (BNPs) that can be fabricated from dye-poly(lactic acid) (PLA) materials [251]. These nanoparticles, on the one hand, have a rigid matrix, and, on the other hand, possess double luminescence: oxygen-sensitive phosphorescence and oxygen-insensitive fluorescence as reference. These spectral properties allow real-time ratiometric determination of oxygen with spatial resolution at micron-level. The probe was tested in different conditions from hypoxic to normoxic and had shown a good response and stability even when increasing K^+^ concentration was used for the neuronal activity stimulation. Thus, such probes could provide fundamental insights into excitability studies and neural mechanisms.

Another recent example of using the ratiometric approach represents a sensor based on a near-infrared (NIR) oxygen-quenched luminophore Pt(II) octaethylporphine ketone (PtOEPK), and a stable reference dye dioctadecyldicarbocyanine (DiD) [252]. The advantage of this nanosensor system is that it provides an approach to overcome problems, such as optical scattering and autofluorescence in the visible wavelength range observed in biological systems. The encapsulation of dyes in a polymer host matrix makes it possible to keep their ratio constant in biological samples and eliminates the need for complex synthetic paths. When the condition of maintaining a constant ratio of the concentration of fluorophores is met, the analytical signal can be represented as the ratio of the intensities of two signals, taking into account the artifacts of the concentration of the nanosensor in the measurements. It is important that the developed nanosensors are reversible, making it possible to perform measurements in systems where the concentration of dissolved oxygen both increases and decreases. The monitoring of *Saccharomyces cerevisiae* (brewing yeast) in a 96-well was performed to evaluate the sensor performance. The nanosensors were added directly to the wells and incubation was monitored by the fluorescence plate reader for 60 h. The system allowed to track metabolic activity and dynamic changes that occur due to the change in cell concentration or because of toxic effects. Thus, it was shown that this system can become a platform for high-throughput screening of various microorganism species with an unknown metabolic rate in response to external stimuli (metabolites, antimicrobials, etc.).

## 5. Conclusions and Prospects

Optical sensing systems have proven to be very powerful in terms of physical scalability, from the tips of optical fibers to a large planar system [253]. However, fluorophore-based sensor films are still limited to a fairly small number of analytes. Thus, synthetics are working hard to expand the range of available sensors. Besides, optical sensors are subject to degradation through photobleaching and fouling. To some extent, these problems can be solved by using replaceable sensing elements, but this only applies to sufficiently large-scale systems, and not to those based on single optical fibers.

The works of recent years presented in this review show a trend towards the use of various fillers forming composites, which, on the one hand, make it possible to increase the fluorescent response, and hence the measurement accuracy, and, on the other hand, to lower the detection limit [108,233].

Another important achievement of composite materials is the possibility of combine in one material either several cooperating molecules, as in the case of the determination of hydrogen peroxide [234,235,238], or several mutually insoluble analytical systems [206,208]. Moreover, new approaches to control wettability and surface biofouling are presented [107,186].

More and more works are published devoted to bioimaging and the study of spatial gradients of various substances during the course of various processes. The measurement principle is based on the determination of the fluorescence lifetime, which is correlated with the phase difference between the excitation light and the fluorescence output. In this case, it is important to take into account the possible nonlinear effects of the fluorescent material, since in this case the accuracy of the results will depend on the modulation frequency of the excitation light [254]. Variation in the modulation frequency showed that it mainly affects the sensitivity and phase resolution ratio of the system. A good degree of fitting with R^2^ value of 0.9981 and a small relative error of 0.79% was observed for the system with the optimal modulation frequency.

It is important to note that, while monitoring of individual substances using optical sensors has proven to be successful; it remains a challenging task to monitor the concentrations of multiple analytes at the same time, especially in mapping and imaging. The main problem is related to the superposition of the emission spectra of dyes, which causes a crosstalk effect of the intensities of the color channels in the recording equipment (RGB or RGB-NIR cameras). However, an original solution has recently been presented that can “free the hands” of experimenters and significantly expand the possibilities for synchronous measurement of concentrations of interest.

Recently, a new approach has been proposed that involves hyperspectral imaging combined with signal deconvolution of overlapping emission spectra of several fluorescent indicator dyes [255]. This method greatly simplifies practical measurements, since reduces the requirements for the absence of overlap in the emission spectra of the dyes used. The deconvolution algorithm to decode the superimposed sensor signals uses a linear combination model. It runs a sequential least-squares fit to determine the contribution of the individual indicator dyes to the total measured signal. As a proof of concept, the algorithm was used to analyze the measured response of an O_2_ sensor composed of red-emitting Pd(II)/Pt(II) porphyrins and NIR-emitting Pd(II)/Pt(II) benzoporphyrins with different sensitivities. The results showed that such a combination allowed imaging of O_2_ over a wide dynamic range (0–950 hPa) with a hyperspectral camera system (470–900 nm). The roots of the aquatic plant *Littorella uniflora* were used to demonstrate the advantage of the novel method by imaging the O_2_ distribution in the heterogeneous microenvironment. The reported approach of combining hyperspectral sensing with signal deconvolution is flexible and can easily be adapted for the use of various multi-indicator- or even multianalyte-based optical sensors with different spectral characteristics, enabling high-resolution simultaneous imaging of multiple analytes.

The combination of described new instrumental approach, and the possibilities presented in this review, achieved by composite polymer-based optical sensors, in our opinion, can significantly enrich our knowledge about the surrounding world in the near future.

## Figures and Tables

**Table 1:** Improvement of the properties of polymer nanocomposites and sensors due to the influence of some nanofillers.

Composite Type	Nanofiller	Sensor Improvement	Ref.
PANI/MNP	MNPs	Magnetic property	[100]
TiO_2_ Nanorod/TiO_2_ Quantum Dot/Polydopamine	Nano rod and Quantum Dot	Strong light absorption and photocatalytic activity	[101]
PVA/CDs	CDs	Ultra-long room temperature phosphorescence, excellent O_2_ diffusion	[102]
OMMT/PLA	Nanoclay	Improved thermal and mechanical property, improved optical properties	[103]
8-HQ/nanoclay epoxy nanocomposite	Nanoclay	Improved adhesion, increased reliability and accuracy of measurements	[104]
Chitosan/AuNPs and Chitosan/AgNPs	Au and Ag NPs	Plasmon resonance of metal nanoparticles improves sensitivity and detection limit	[105]
Fluorinated polymer/SiO_2_	SiO_2_ core with adsorbed dye and fluorinated shell	Calibration curve linearization, dye leaching protection, biofouling resistance	[106]
Fluorinated polymer/DND	DND	Tunable surface adhesion, protection/improvement of biomaterial adhesion	[107]
Polymer/SiO_2_/AuNPs	SiO_2_ with smaller AuNPs	Luminescence enhancement, tunable plasmonic resonance peak	[108]
PANI nanofibers/MOF	MOF	Suppressed aggregation of dye molecules, highest oxygen permeability known	[109]

PANI: Polyaniline, MNP: Magnetic nanoparticle, PVA: polyvinyl alcohol, CDs: carbon dots, OMMT: Organically modified montmorillonite, PLA: Polylactide, 8-HQ: 8-hydroxyquinoline, AuNPs and AgNPs: Au and Ag nanoparticles, DND: modified detonation nanodiamonds, MOF: Metal−organic framework.

## Data Availability

Not applicable.
